# Bis(5,5-diphenyl­hydantoinato-κ*N* ^3^)(ethyl­enediamine)zinc(II)

**DOI:** 10.1107/S160053680904313X

**Published:** 2009-10-23

**Authors:** Xilan Hu, Xingyou Xu, Daqi Wang, Yu Zhang

**Affiliations:** a Huaihai Institute of Technology, Jiangsu 222005, People’s Republic of China; b Huaiyin Institute of Technology, Jiangsu 223003, People’s Republic of China; c College of Chemistry and Chemical Engineering, Liaocheng University, Shandong 252059, People’s Republic of China

## Abstract

In the title compound, [Zn(C_15_H_11_N_2_O_2_)_2_(C_2_H_8_N_2_)], the Zn^II^ atom is coordinated in a distorted tetra­hedral geometry. Intra­molecular N—H⋯O, C—H⋯O and C—H⋯N hydrogen bonds occur. In the crystal, mol­ecules are linked by inter­molecular N—H⋯O hydrogen bonds, forming a three-dimensional network.

## Related literature

5,5-Diphenyl­imidazoline-2,4-dione (phenytoin) is widely used in the treatment of epilepsy and should be an excellent ligand for transition metal complexes, see: Milne *et al.* (1999 ▶); Akitsu & Einaga (2005 ▶); Akitsu *et al.* (1997 ▶). For complexes with 5,5-diphenyl­hydantoinate, see: Hu, Xu, Wang & Xu (2006 ▶); Hu, Xu, Xu & Wang (2006 ▶).
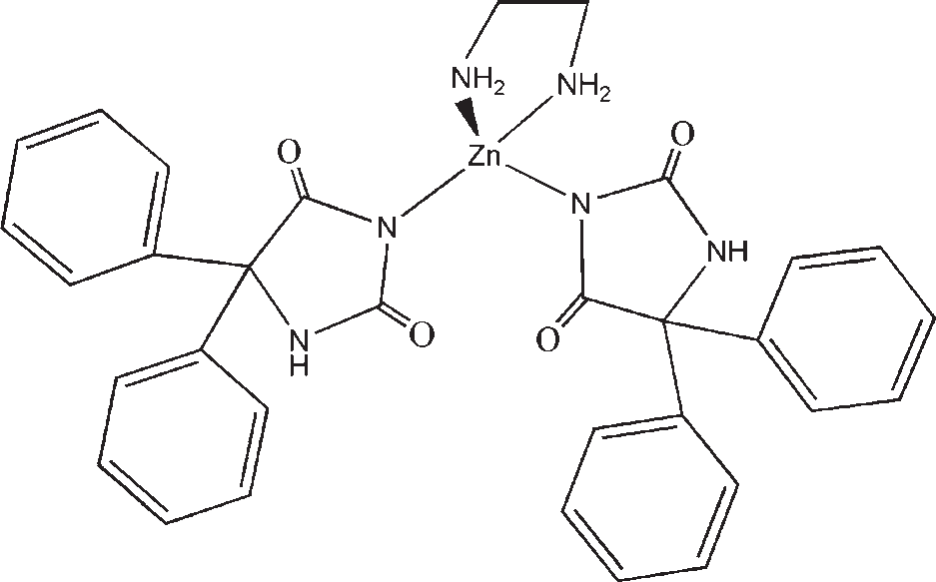


## Experimental

### 

#### Crystal data

[Zn(C_15_H_11_N_2_O_2_)_2_(C_2_H_8_N_2_)]
*M* *_r_* = 627.99
Triclinic, 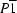

*a* = 9.702 (1) Å
*b* = 13.052 (2) Å
*c* = 13.293 (2) Å
α = 109.114 (2)°
β = 109.462 (2)°
γ = 93.020(10)°
*V* = 1473.1 (3) Å^3^
*Z* = 2
Mo *K*α radiation
μ = 0.88 mm^−1^
*T* = 298 K
0.50 × 0.46 × 0.32 mm

#### Data collection

Bruker SMART CCD area-detector diffractometer
Absorption correction: multi-scan (*SADABS*; Sheldrick, 1996 ▶) *T*_min_ = 0.667, *T*_max_ = 0.766
7725 measured reflections
5124 independent reflections
4452 reflections with *I* > 2σ(*I*)
*R*_int_ = 0.017

#### Refinement

*R*[*F* ^2^ > 2σ(*F* ^2^)] = 0.034
*wR*(*F* ^2^) = 0.089
*S* = 1.03
5124 reflections
388 parameters
H-atom parameters constrained
Δρ_max_ = 0.61 e Å^−3^
Δρ_min_ = −0.45 e Å^−3^

### 

Data collection: *SMART* (Siemens, 1996 ▶); cell refinement: *SAINT* (Siemens, 1996 ▶); data reduction: *SAINT*; program(s) used to solve structure: *SHELXS97* (Sheldrick, 2008 ▶); program(s) used to refine structure: *SHELXL97* (Sheldrick, 2008 ▶); molecular graphics: *SHELXTL* (Sheldrick, 2008 ▶); software used to prepare material for publication: *SHELXTL*.

## Supplementary Material

Crystal structure: contains datablocks I, global. DOI: 10.1107/S160053680904313X/bx2240sup1.cif

Structure factors: contains datablocks I. DOI: 10.1107/S160053680904313X/bx2240Isup2.hkl

Additional supplementary materials:  crystallographic information; 3D view; checkCIF report

## Figures and Tables

**Table 1:** Hydrogen-bond geometry (Å, °)

*D*—H⋯*A*	*D*—H	H⋯*A*	*D*⋯*A*	*D*—H⋯*A*
N6—H6*B*⋯O2	0.90	2.18	2.858 (3)	132
C9—H9⋯O2	0.93	2.36	2.992 (3)	125
C11—H11⋯N1	0.93	2.52	2.860 (4)	102
C24—H24⋯O4	0.93	2.39	3.042 (4)	127
C26—H26⋯N3	0.93	2.51	2.854 (4)	102
N1—H1⋯O1^i^	0.86	2.16	3.008 (3)	167
N3—H3⋯O3^ii^	0.86	2.04	2.843 (3)	156
N5—H5*A*⋯O4^iii^	0.90	1.97	2.855 (3)	170
N5—H5*B*⋯O1^iii^	0.90	2.18	3.003 (3)	152

Symmetry codes: (i) 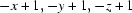; (ii) 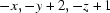; (iii) 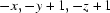.

**Fig. 1.**
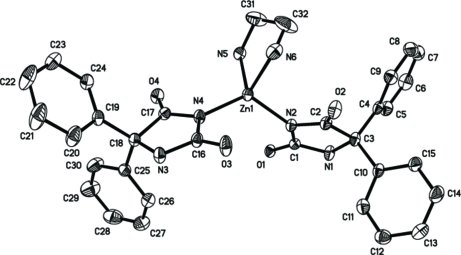
The molecular structure of the title complex. Displacement ellipsoids are drawn at the 30% probability level. The H-atom have been omitted for clarity.

## supplementary crystallographic information

### Comment

5,5-diphenylimidazoline-2,4-dione (phenytoin) compound is a widely used drug in the treatment of epilepsy and should be an excellent ligand for transition metal complex (Milne *et al.*,  1999; Akitsu, Komorita, Kushi *et al.*,1997; Akitsu, Einaga, 2005). We have designed and synthesized a series of complexes with 5,5-diphenylhydantoinate (Hu, Xu, Wang *et al.*, 2006). We report here the crystal structure of the title compound (I). The compound (Fig. 1) consists of [Zn(pht)_2_(en)] (pht=5,5-diphenylhydantoinato; en=ethylendiamine) complex neutral molecule. The Zn atom is coordinated by two nitrogen atoms from two pht ligands and two nitrogen atoms from two en ligands and is in a distorted tetrahedron ZnN_4_ coordination environment.The Zn—N bond distances lie in the range of 1.9506 (18)Å to 2.057 (2) Å. There are intra- and intermolecular N—H···O=C hydrogen bonds, forming a three-dimensional network in the crystal structure, Table 1.

### Experimental

To a solution of pht (1.00 mmol) in methanol (10 ml) was added zinc(II) acetate tetrahydrate (0.5 mmol) and the solution of Ethylenediamine(0.5 mmol) in methanol (10 ml). Then the mixture was sealed in a 25 ml stainless steel vessel with Teflon linear and heated to 393 K for 50 h the fill rate being 80%. After cooling to room temperature, the colorless single crystals were obtained by slow evaporation from the filtrate.

### Refinement

The space group was uniquely assigned from the systematic absences. All H atoms were placed at calculated positions, with N—H = 0.86–0.90 Å, and with *U*_iso_(H) values were set at 1.2*U*_eq_, and C—H = 0.93Å (phenyl), 0.97Å (methylene), respectively, and with *U*_iso_(H) values were set at 1.2 *U*_eq_(C) (phenyl,methylene).

### Crystal data

**Table:**

[Zn(C_15_H_11_N_2_O_2_)_2_(C_2_H_8_N_2_)]	*Z* = 2
*M**_r_* = 627.99	*F*(000) = 652
Triclinic, *P*1	*D*_x_ = 1.416 Mg m^−^^3^
Hall symbol: -P 1	Mo *K*α radiation, λ = 0.71073 Å
*a* = 9.702 (1) Å	Cell parameters from 4727 reflections
*b* = 13.0520 (15) Å	θ = 2.6–27.7°
*c* = 13.2930 (16) Å	µ = 0.88 mm^−^^1^
α = 109.114 (2)°	*T* = 298 K
β = 109.462 (2)°	Block, colorless
γ = 93.302 (1)°	0.50 × 0.46 × 0.32 mm
*V* = 1473.1 (3) Å^3^	

### Data collection

**Table:**

Bruker SMART CCD area-detector diffractometer	5124 independent reflections
Radiation source: fine-focus sealed tube	4452 reflections with *I* > 2σ(*I*)
graphite	*R*_int_ = 0.017
φ and ω scans	θ_max_ = 25.0°, θ_min_ = 1.7°
Absorption correction: multi-scan (*SADABS*; Sheldrick, 1996)	*h* = −11→11
*T*_min_ = 0.667, *T*_max_ = 0.766	*k* = −15→15
7725 measured reflections	*l* = −14→15

### Refinement

**Table:**

Refinement on *F*^2^	Primary atom site location: structure-invariant direct methods
Least-squares matrix: full	Secondary atom site location: difference Fourier map
*R*[*F*^2^ > 2σ(*F*^2^)] = 0.034	Hydrogen site location: inferred from neighbouring sites
*wR*(*F*^2^) = 0.089	H-atom parameters constrained
*S* = 1.03	*w* = 1/[σ^2^(*F*_o_^2^) + (0.0432*P*)^2^ + 0.9132*P*] where *P* = (*F*_o_^2^ + 2*F*_c_^2^)/3
5124 reflections	(Δ/σ)_max_ = 0.001
388 parameters	Δρ_max_ = 0.61 e Å^−^^3^
0 restraints	Δρ_min_ = −0.45 e Å^−^^3^

### Special details

**Table:**

Geometry. All e.s.d.'s (except the e.s.d. in the dihedral angle between two l.s. planes) are estimated using the full covariance matrix. The cell e.s.d.'s are taken into account individually in the estimation of e.s.d.'s in distances, angles and torsion angles; correlations between e.s.d.'s in cell parameters are only used when they are defined by crystal symmetry. An approximate (isotropic) treatment of cell e.s.d.'s is used for estimating e.s.d.'s involving l.s. planes.
Refinement. Refinement of *F*^2^ against ALL reflections. The weighted *R*-factor *wR* and goodness of fit *S* are based on *F*^2^, conventional *R*-factors *R* are based on *F*, with *F* set to zero for negative *F*^2^. The threshold expression of *F*^2^ > σ(*F*^2^) is used only for calculating *R*-factors(gt) *etc*. and is not relevant to the choice of reflections for refinement. *R*-factors based on *F*^2^ are statistically about twice as large as those based on *F*, and *R*- factors based on ALL data will be even larger.

### Fractional atomic coordinates and isotropic or equivalent isotropic displacement parameters (Å^2^)

**Table:**

	*x*	*y*	*z*	*U*_iso_*/*U*_eq_	
Zn1	−0.00468 (3)	0.61290 (2)	0.37871 (2)	0.02823 (10)	
N1	0.4053 (2)	0.53299 (18)	0.37370 (16)	0.0358 (5)	
H1	0.4894	0.5154	0.4032	0.043*	
N2	0.1898 (2)	0.59269 (15)	0.36211 (16)	0.0290 (4)	
N3	−0.0141 (3)	0.92085 (15)	0.58602 (18)	0.0380 (5)	
H3	0.0040	0.9910	0.6028	0.046*	
N4	−0.0279 (2)	0.74051 (15)	0.49503 (17)	0.0328 (4)	
N5	−0.1098 (2)	0.46617 (15)	0.36800 (17)	0.0335 (4)	
H5A	−0.0432	0.4228	0.3835	0.040*	
H5B	−0.1548	0.4787	0.4190	0.040*	
N6	−0.1472 (2)	0.5694 (2)	0.21093 (19)	0.0480 (6)	
H6A	−0.2288	0.5998	0.2067	0.058*	
H6B	−0.1020	0.5906	0.1699	0.058*	
O1	0.33097 (18)	0.56196 (14)	0.52547 (13)	0.0337 (4)	
O2	0.1300 (2)	0.61465 (16)	0.18764 (15)	0.0455 (5)	
O3	0.0303 (2)	0.86389 (14)	0.41865 (17)	0.0510 (5)	
O4	−0.1104 (2)	0.66928 (13)	0.60608 (16)	0.0401 (4)	
C1	0.3111 (2)	0.56151 (18)	0.42891 (19)	0.0278 (5)	
C2	0.2089 (3)	0.5866 (2)	0.2636 (2)	0.0318 (5)	
C3	0.3484 (3)	0.5354 (2)	0.25864 (19)	0.0319 (5)	
C4	0.2992 (3)	0.4187 (2)	0.1657 (2)	0.0333 (5)	
C5	0.3729 (3)	0.3350 (2)	0.1842 (2)	0.0433 (6)	
H5	0.4527	0.3499	0.2528	0.052*	
C6	0.3292 (4)	0.2292 (2)	0.1016 (3)	0.0537 (7)	
H6	0.3807	0.1741	0.1151	0.064*	
C7	0.2103 (4)	0.2046 (2)	−0.0001 (3)	0.0523 (7)	
H7	0.1812	0.1334	−0.0550	0.063*	
C8	0.1354 (3)	0.2864 (2)	−0.0196 (2)	0.0498 (7)	
H8	0.0545	0.2705	−0.0878	0.060*	
C9	0.1796 (3)	0.3926 (2)	0.0618 (2)	0.0420 (6)	
H9	0.1288	0.4476	0.0470	0.050*	
C10	0.4530 (3)	0.6143 (2)	0.2406 (2)	0.0325 (5)	
C11	0.5446 (3)	0.7065 (2)	0.3331 (2)	0.0440 (6)	
H11	0.5478	0.7163	0.4064	0.053*	
C12	0.6311 (3)	0.7837 (2)	0.3181 (3)	0.0490 (7)	
H12	0.6919	0.8447	0.3811	0.059*	
C13	0.6278 (3)	0.7708 (2)	0.2107 (3)	0.0500 (7)	
H13	0.6852	0.8233	0.2006	0.060*	
C14	0.5390 (3)	0.6797 (3)	0.1181 (3)	0.0553 (8)	
H14	0.5364	0.6706	0.0451	0.066*	
C15	0.4527 (3)	0.6011 (2)	0.1331 (2)	0.0469 (7)	
H15	0.3943	0.5391	0.0701	0.056*	
C16	−0.0009 (3)	0.84551 (19)	0.4940 (2)	0.0346 (6)	
C17	−0.0734 (3)	0.74627 (18)	0.5818 (2)	0.0303 (5)	
C18	−0.0629 (3)	0.86969 (18)	0.6538 (2)	0.0313 (5)	
C19	−0.2101 (3)	0.89940 (19)	0.6608 (2)	0.0341 (5)	
C20	−0.2238 (4)	1.0100 (2)	0.6886 (3)	0.0608 (9)	
H20	−0.1437	1.0630	0.7039	0.073*	
C21	−0.3546 (5)	1.0409 (3)	0.6938 (4)	0.0841 (13)	
H21	−0.3623	1.1150	0.7134	0.101*	
C22	−0.4754 (4)	0.9628 (3)	0.6699 (4)	0.0769 (11)	
H22	−0.5651	0.9839	0.6709	0.092*	
C23	−0.4618 (3)	0.8539 (3)	0.6447 (3)	0.0568 (8)	
H23	−0.5422	0.8011	0.6295	0.068*	
C24	−0.3285 (3)	0.8228 (2)	0.6420 (2)	0.0422 (6)	
H24	−0.3190	0.7494	0.6274	0.051*	
C25	0.0588 (3)	0.89167 (19)	0.7714 (2)	0.0357 (6)	
C26	0.2070 (3)	0.9131 (2)	0.7872 (3)	0.0530 (7)	
H26	0.2329	0.9217	0.7286	0.064*	
C27	0.3172 (4)	0.9218 (3)	0.8887 (3)	0.0681 (10)	
H27	0.4166	0.9367	0.8982	0.082*	
C28	0.2816 (4)	0.9088 (3)	0.9754 (3)	0.0697 (10)	
H28	0.3562	0.9137	1.0433	0.084*	
C29	0.1353 (4)	0.8884 (3)	0.9616 (3)	0.0718 (10)	
H29	0.1104	0.8803	1.0208	0.086*	
C30	0.0239 (3)	0.8797 (3)	0.8599 (3)	0.0554 (8)	
H30	−0.0753	0.8657	0.8512	0.066*	
C31	−0.2203 (4)	0.4122 (3)	0.2507 (3)	0.0715 (10)	
H31A	−0.2261	0.3330	0.2275	0.086*	
H31B	−0.3172	0.4277	0.2499	0.086*	
C32	−0.1855 (5)	0.4482 (3)	0.1696 (3)	0.0890 (14)	
H32A	−0.2703	0.4216	0.0975	0.107*	
H32B	−0.1024	0.4167	0.1557	0.107*	

### Atomic displacement parameters (Å^2^)

**Table:**

	*U*^11^	*U*^22^	*U*^33^	*U*^12^	*U*^13^	*U*^23^
Zn1	0.03145 (16)	0.02810 (15)	0.03137 (16)	0.01168 (11)	0.01542 (12)	0.01390 (11)
N1	0.0317 (11)	0.0594 (13)	0.0302 (11)	0.0233 (10)	0.0153 (9)	0.0273 (10)
N2	0.0279 (10)	0.0367 (10)	0.0280 (10)	0.0121 (8)	0.0125 (8)	0.0160 (8)
N3	0.0587 (14)	0.0218 (9)	0.0486 (13)	0.0109 (9)	0.0358 (11)	0.0151 (9)
N4	0.0423 (12)	0.0242 (9)	0.0404 (11)	0.0116 (9)	0.0239 (10)	0.0127 (9)
N5	0.0362 (11)	0.0334 (10)	0.0375 (11)	0.0081 (9)	0.0163 (9)	0.0185 (9)
N6	0.0366 (13)	0.0734 (16)	0.0464 (13)	0.0160 (12)	0.0117 (11)	0.0402 (13)
O1	0.0337 (9)	0.0471 (10)	0.0257 (8)	0.0093 (7)	0.0127 (7)	0.0183 (7)
O2	0.0418 (11)	0.0743 (13)	0.0428 (10)	0.0326 (10)	0.0212 (9)	0.0405 (10)
O3	0.0859 (15)	0.0372 (10)	0.0595 (12)	0.0229 (10)	0.0542 (12)	0.0252 (9)
O4	0.0506 (11)	0.0293 (9)	0.0547 (11)	0.0117 (8)	0.0299 (9)	0.0222 (8)
C1	0.0269 (12)	0.0297 (11)	0.0285 (12)	0.0061 (9)	0.0102 (10)	0.0128 (10)
C2	0.0284 (12)	0.0412 (13)	0.0326 (13)	0.0129 (10)	0.0130 (10)	0.0193 (11)
C3	0.0291 (13)	0.0496 (14)	0.0273 (12)	0.0168 (11)	0.0129 (10)	0.0231 (11)
C4	0.0311 (13)	0.0462 (14)	0.0347 (13)	0.0105 (11)	0.0181 (11)	0.0232 (11)
C5	0.0392 (15)	0.0525 (16)	0.0471 (15)	0.0186 (13)	0.0187 (13)	0.0253 (13)
C6	0.061 (2)	0.0490 (17)	0.066 (2)	0.0235 (15)	0.0309 (17)	0.0291 (15)
C7	0.064 (2)	0.0444 (16)	0.0512 (17)	0.0022 (14)	0.0278 (16)	0.0163 (14)
C8	0.0516 (18)	0.0568 (17)	0.0396 (15)	0.0017 (14)	0.0126 (13)	0.0219 (14)
C9	0.0432 (16)	0.0499 (16)	0.0390 (14)	0.0127 (12)	0.0145 (12)	0.0240 (13)
C10	0.0271 (12)	0.0461 (14)	0.0335 (13)	0.0159 (11)	0.0146 (10)	0.0213 (11)
C11	0.0457 (16)	0.0524 (16)	0.0381 (14)	0.0149 (13)	0.0185 (13)	0.0181 (13)
C12	0.0421 (16)	0.0460 (16)	0.0527 (17)	0.0083 (13)	0.0136 (13)	0.0146 (13)
C13	0.0414 (16)	0.0540 (17)	0.067 (2)	0.0096 (13)	0.0243 (15)	0.0329 (16)
C14	0.0559 (19)	0.073 (2)	0.0485 (17)	0.0045 (16)	0.0241 (15)	0.0330 (16)
C15	0.0444 (16)	0.0629 (18)	0.0347 (14)	−0.0003 (13)	0.0147 (12)	0.0215 (13)
C16	0.0427 (15)	0.0273 (12)	0.0445 (14)	0.0128 (10)	0.0267 (12)	0.0148 (11)
C17	0.0305 (13)	0.0267 (11)	0.0373 (13)	0.0088 (10)	0.0148 (10)	0.0134 (10)
C18	0.0386 (14)	0.0249 (11)	0.0377 (13)	0.0078 (10)	0.0212 (11)	0.0131 (10)
C19	0.0419 (14)	0.0357 (13)	0.0344 (13)	0.0146 (11)	0.0207 (11)	0.0171 (11)
C20	0.068 (2)	0.0454 (16)	0.101 (3)	0.0266 (15)	0.057 (2)	0.0371 (17)
C21	0.094 (3)	0.067 (2)	0.153 (4)	0.055 (2)	0.088 (3)	0.067 (3)
C22	0.065 (2)	0.093 (3)	0.123 (3)	0.050 (2)	0.063 (2)	0.069 (3)
C23	0.0432 (17)	0.071 (2)	0.068 (2)	0.0131 (15)	0.0288 (16)	0.0306 (17)
C24	0.0461 (16)	0.0405 (14)	0.0478 (16)	0.0121 (12)	0.0248 (13)	0.0179 (12)
C25	0.0374 (14)	0.0278 (12)	0.0418 (14)	0.0053 (10)	0.0180 (12)	0.0096 (11)
C26	0.0434 (17)	0.0584 (18)	0.0517 (17)	0.0047 (14)	0.0224 (14)	0.0095 (14)
C27	0.0370 (17)	0.078 (2)	0.069 (2)	0.0039 (16)	0.0139 (16)	0.0100 (19)
C28	0.055 (2)	0.070 (2)	0.060 (2)	0.0040 (17)	−0.0032 (17)	0.0200 (18)
C29	0.062 (2)	0.100 (3)	0.0502 (19)	−0.002 (2)	0.0093 (17)	0.0381 (19)
C30	0.0393 (16)	0.080 (2)	0.0496 (17)	0.0003 (15)	0.0144 (14)	0.0311 (16)
C31	0.078 (2)	0.059 (2)	0.053 (2)	−0.0192 (18)	0.0014 (18)	0.0198 (16)
C32	0.110 (3)	0.081 (3)	0.0382 (18)	−0.027 (2)	−0.0048 (19)	0.0157 (18)

### Geometric parameters (Å, °)

**Table:**

Zn1—N4	1.9506 (18)	C11—C12	1.381 (4)
Zn1—N2	1.9941 (19)	C11—H11	0.9300
Zn1—N5	2.0561 (19)	C12—C13	1.370 (4)
Zn1—N6	2.057 (2)	C12—H12	0.9300
N1—C1	1.349 (3)	C13—C14	1.374 (4)
N1—C3	1.455 (3)	C13—H13	0.9300
N1—H1	0.8600	C14—C15	1.392 (4)
N2—C2	1.360 (3)	C14—H14	0.9300
N2—C1	1.390 (3)	C15—H15	0.9300
N3—C16	1.346 (3)	C17—C18	1.558 (3)
N3—C18	1.457 (3)	C18—C19	1.525 (3)
N3—H3	0.8600	C18—C25	1.535 (4)
N4—C17	1.348 (3)	C19—C24	1.377 (4)
N4—C16	1.386 (3)	C19—C20	1.394 (4)
N5—C31	1.469 (4)	C20—C21	1.370 (4)
N5—H5A	0.9000	C20—H20	0.9300
N5—H5B	0.9000	C21—C22	1.384 (5)
N6—C32	1.472 (4)	C21—H21	0.9300
N6—H6A	0.9000	C22—C23	1.374 (5)
N6—H6B	0.9000	C22—H22	0.9300
O1—C1	1.231 (3)	C23—C24	1.386 (4)
O2—C2	1.224 (3)	C23—H23	0.9300
O3—C16	1.226 (3)	C24—H24	0.9300
O4—C17	1.221 (3)	C25—C30	1.379 (4)
C2—C3	1.555 (3)	C25—C26	1.380 (4)
C3—C10	1.534 (3)	C26—C27	1.379 (5)
C3—C4	1.537 (3)	C26—H26	0.9300
C4—C5	1.384 (3)	C27—C28	1.365 (5)
C4—C9	1.396 (4)	C27—H27	0.9300
C5—C6	1.386 (4)	C28—C29	1.368 (5)
C5—H5	0.9300	C28—H28	0.9300
C6—C7	1.377 (4)	C29—C30	1.386 (4)
C6—H6	0.9300	C29—H29	0.9300
C7—C8	1.371 (4)	C30—H30	0.9300
C7—H7	0.9300	C31—C32	1.433 (5)
C8—C9	1.384 (4)	C31—H31A	0.9700
C8—H8	0.9300	C31—H31B	0.9700
C9—H9	0.9300	C32—H32A	0.9700
C10—C15	1.382 (3)	C32—H32B	0.9700
C10—C11	1.389 (4)		
			
N4—Zn1—N2	123.12 (8)	C12—C13—C14	119.5 (3)
N4—Zn1—N5	112.66 (8)	C12—C13—H13	120.2
N2—Zn1—N5	108.94 (8)	C14—C13—H13	120.2
N4—Zn1—N6	118.42 (9)	C13—C14—C15	120.3 (3)
N2—Zn1—N6	102.08 (9)	C13—C14—H14	119.8
N5—Zn1—N6	84.83 (9)	C15—C14—H14	119.8
C1—N1—C3	112.29 (18)	C10—C15—C14	120.7 (3)
C1—N1—H1	123.9	C10—C15—H15	119.7
C3—N1—H1	123.9	C14—C15—H15	119.7
C2—N2—C1	108.39 (18)	O3—C16—N3	126.6 (2)
C2—N2—Zn1	120.78 (15)	O3—C16—N4	123.3 (2)
C1—N2—Zn1	129.63 (15)	N3—C16—N4	110.1 (2)
C16—N3—C18	112.08 (19)	O4—C17—N4	126.9 (2)
C16—N3—H3	124.0	O4—C17—C18	123.8 (2)
C18—N3—H3	124.0	N4—C17—C18	109.18 (18)
C17—N4—C16	109.22 (18)	N3—C18—C19	111.23 (19)
C17—N4—Zn1	130.24 (15)	N3—C18—C25	112.6 (2)
C16—N4—Zn1	120.51 (16)	C19—C18—C25	113.46 (19)
C31—N5—Zn1	107.60 (17)	N3—C18—C17	99.11 (17)
C31—N5—H5A	110.2	C19—C18—C17	114.35 (19)
Zn1—N5—H5A	110.2	C25—C18—C17	105.10 (18)
C31—N5—H5B	110.2	C24—C19—C20	118.8 (2)
Zn1—N5—H5B	110.2	C24—C19—C18	123.3 (2)
H5A—N5—H5B	108.5	C20—C19—C18	117.9 (2)
C32—N6—Zn1	103.62 (18)	C21—C20—C19	120.3 (3)
C32—N6—H6A	111.0	C21—C20—H20	119.8
Zn1—N6—H6A	111.0	C19—C20—H20	119.8
C32—N6—H6B	111.0	C20—C21—C22	120.6 (3)
Zn1—N6—H6B	111.0	C20—C21—H21	119.7
H6A—N6—H6B	109.0	C22—C21—H21	119.7
O1—C1—N1	124.9 (2)	C23—C22—C21	119.5 (3)
O1—C1—N2	124.9 (2)	C23—C22—H22	120.3
N1—C1—N2	110.25 (19)	C21—C22—H22	120.3
O2—C2—N2	127.1 (2)	C22—C23—C24	120.0 (3)
O2—C2—C3	123.4 (2)	C22—C23—H23	120.0
N2—C2—C3	109.58 (18)	C24—C23—H23	120.0
N1—C3—C10	112.7 (2)	C19—C24—C23	120.7 (3)
N1—C3—C4	111.98 (19)	C19—C24—H24	119.7
C10—C3—C4	114.11 (18)	C23—C24—H24	119.7
N1—C3—C2	98.85 (17)	C30—C25—C26	118.3 (3)
C10—C3—C2	108.59 (19)	C30—C25—C18	121.1 (2)
C4—C3—C2	109.47 (19)	C26—C25—C18	120.3 (2)
C5—C4—C9	117.7 (2)	C27—C26—C25	120.7 (3)
C5—C4—C3	120.1 (2)	C27—C26—H26	119.6
C9—C4—C3	122.2 (2)	C25—C26—H26	119.6
C4—C5—C6	120.8 (3)	C28—C27—C26	120.5 (3)
C4—C5—H5	119.6	C28—C27—H27	119.7
C6—C5—H5	119.6	C26—C27—H27	119.7
C7—C6—C5	120.8 (3)	C27—C28—C29	119.5 (3)
C7—C6—H6	119.6	C27—C28—H28	120.2
C5—C6—H6	119.6	C29—C28—H28	120.2
C8—C7—C6	119.3 (3)	C28—C29—C30	120.3 (3)
C8—C7—H7	120.4	C28—C29—H29	119.9
C6—C7—H7	120.4	C30—C29—H29	119.9
C7—C8—C9	120.3 (3)	C25—C30—C29	120.6 (3)
C7—C8—H8	119.8	C25—C30—H30	119.7
C9—C8—H8	119.8	C29—C30—H30	119.7
C8—C9—C4	121.1 (3)	C32—C31—N5	112.7 (3)
C8—C9—H9	119.4	C32—C31—H31A	109.1
C4—C9—H9	119.4	N5—C31—H31A	109.1
C15—C10—C11	118.0 (2)	C32—C31—H31B	109.1
C15—C10—C3	122.0 (2)	N5—C31—H31B	109.1
C11—C10—C3	119.8 (2)	H31A—C31—H31B	107.8
C12—C11—C10	121.1 (3)	C31—C32—N6	111.7 (3)
C12—C11—H11	119.4	C31—C32—H32A	109.3
C10—C11—H11	119.4	N6—C32—H32A	109.3
C13—C12—C11	120.3 (3)	C31—C32—H32B	109.3
C13—C12—H12	119.8	N6—C32—H32B	109.3
C11—C12—H12	119.8	H32A—C32—H32B	107.9
			
N4—Zn1—N2—C2	−118.65 (18)	C3—C10—C11—C12	−174.7 (2)
N5—Zn1—N2—C2	106.10 (18)	C10—C11—C12—C13	0.2 (4)
N6—Zn1—N2—C2	17.5 (2)	C11—C12—C13—C14	−0.8 (4)
N4—Zn1—N2—C1	75.4 (2)	C12—C13—C14—C15	0.1 (5)
N5—Zn1—N2—C1	−59.8 (2)	C11—C10—C15—C14	−1.7 (4)
N6—Zn1—N2—C1	−148.45 (19)	C3—C10—C15—C14	174.0 (3)
N2—Zn1—N4—C17	−118.7 (2)	C13—C14—C15—C10	1.1 (5)
N5—Zn1—N4—C17	15.1 (2)	C18—N3—C16—O3	175.9 (3)
N6—Zn1—N4—C17	111.7 (2)	C18—N3—C16—N4	−4.2 (3)
N2—Zn1—N4—C16	63.7 (2)	C17—N4—C16—O3	−174.0 (3)
N5—Zn1—N4—C16	−162.46 (18)	Zn1—N4—C16—O3	4.0 (4)
N6—Zn1—N4—C16	−65.9 (2)	C17—N4—C16—N3	6.1 (3)
N4—Zn1—N5—C31	120.3 (2)	Zn1—N4—C16—N3	−175.88 (17)
N2—Zn1—N5—C31	−99.4 (2)	C16—N4—C17—O4	178.0 (2)
N6—Zn1—N5—C31	1.6 (2)	Zn1—N4—C17—O4	0.2 (4)
N4—Zn1—N6—C32	−137.5 (2)	C16—N4—C17—C18	−5.5 (3)
N2—Zn1—N6—C32	83.8 (2)	Zn1—N4—C17—C18	176.76 (16)
N5—Zn1—N6—C32	−24.5 (2)	C16—N3—C18—C19	−119.9 (2)
C3—N1—C1—O1	177.2 (2)	C16—N3—C18—C25	111.5 (2)
C3—N1—C1—N2	−4.1 (3)	C16—N3—C18—C17	0.8 (3)
C2—N2—C1—O1	177.2 (2)	O4—C17—C18—N3	179.6 (2)
Zn1—N2—C1—O1	−15.6 (3)	N4—C17—C18—N3	2.9 (2)
C2—N2—C1—N1	−1.5 (3)	O4—C17—C18—C19	−62.1 (3)
Zn1—N2—C1—N1	165.76 (16)	N4—C17—C18—C19	121.2 (2)
C1—N2—C2—O2	−174.8 (2)	O4—C17—C18—C25	63.0 (3)
Zn1—N2—C2—O2	16.6 (4)	N4—C17—C18—C25	−113.7 (2)
C1—N2—C2—C3	6.1 (3)	N3—C18—C19—C24	132.8 (2)
Zn1—N2—C2—C3	−162.49 (15)	C25—C18—C19—C24	−99.0 (3)
C1—N1—C3—C10	121.6 (2)	C17—C18—C19—C24	21.6 (3)
C1—N1—C3—C4	−108.2 (2)	N3—C18—C19—C20	−47.8 (3)
C1—N1—C3—C2	7.1 (3)	C25—C18—C19—C20	80.4 (3)
O2—C2—C3—N1	173.0 (2)	C17—C18—C19—C20	−159.1 (2)
N2—C2—C3—N1	−7.9 (2)	C24—C19—C20—C21	−1.9 (5)
O2—C2—C3—C10	55.4 (3)	C18—C19—C20—C21	178.7 (3)
N2—C2—C3—C10	−125.5 (2)	C19—C20—C21—C22	−0.9 (6)
O2—C2—C3—C4	−69.8 (3)	C20—C21—C22—C23	2.2 (7)
N2—C2—C3—C4	109.3 (2)	C21—C22—C23—C24	−0.8 (6)
N1—C3—C4—C5	−32.8 (3)	C20—C19—C24—C23	3.4 (4)
C10—C3—C4—C5	96.7 (3)	C18—C19—C24—C23	−177.2 (2)
C2—C3—C4—C5	−141.3 (2)	C22—C23—C24—C19	−2.1 (5)
N1—C3—C4—C9	146.4 (2)	N3—C18—C25—C30	160.6 (2)
C10—C3—C4—C9	−84.1 (3)	C19—C18—C25—C30	33.1 (3)
C2—C3—C4—C9	37.8 (3)	C17—C18—C25—C30	−92.6 (3)
C9—C4—C5—C6	0.3 (4)	N3—C18—C25—C26	−26.0 (3)
C3—C4—C5—C6	179.5 (2)	C19—C18—C25—C26	−153.5 (2)
C4—C5—C6—C7	−0.8 (4)	C17—C18—C25—C26	80.9 (3)
C5—C6—C7—C8	0.4 (4)	C30—C25—C26—C27	0.2 (4)
C6—C7—C8—C9	0.5 (4)	C18—C25—C26—C27	−173.4 (3)
C7—C8—C9—C4	−1.0 (4)	C25—C26—C27—C28	0.4 (5)
C5—C4—C9—C8	0.6 (4)	C26—C27—C28—C29	−0.9 (6)
C3—C4—C9—C8	−178.6 (2)	C27—C28—C29—C30	0.8 (6)
N1—C3—C10—C15	155.5 (2)	C26—C25—C30—C29	−0.4 (5)
C4—C3—C10—C15	26.3 (3)	C18—C25—C30—C29	173.2 (3)
C2—C3—C10—C15	−96.1 (3)	C28—C29—C30—C25	−0.1 (6)
N1—C3—C10—C11	−28.9 (3)	Zn1—N5—C31—C32	23.6 (4)
C4—C3—C10—C11	−158.1 (2)	N5—C31—C32—N6	−47.9 (5)
C2—C3—C10—C11	79.5 (3)	Zn1—N6—C32—C31	45.3 (4)
C15—C10—C11—C12	1.0 (4)		

### Hydrogen-bond geometry (Å, °)

**Table:**

*D*—H···*A*	*D*—H	H···*A*	*D*···*A*	*D*—H···*A*
N6—H6B···O2	0.90	2.18	2.858 (3)	132
C9—H9···O2	0.93	2.36	2.992 (3)	125
C11—H11···N1	0.93	2.52	2.860 (4)	102
C24—H24···O4	0.93	2.39	3.042 (4)	127
C26—H26···N3	0.93	2.51	2.854 (4)	102
N1—H1···O1^i^	0.86	2.16	3.008 (3)	167
N3—H3···O3^ii^	0.86	2.04	2.843 (3)	156
N5—H5A···O4^iii^	0.90	1.97	2.855 (3)	170
N5—H5B···O1^iii^	0.90	2.18	3.003 (3)	152

Symmetry codes: (i) −*x*+1, −*y*+1, −*z*+1; (ii) −*x*, −*y*+2, −*z*+1; (iii) −*x*, −*y*+1, −*z*+1.

## References

[bb1] Akitsu, T. & Einaga, Y. (2005). *Acta Cryst.* C**61**, m183–m186. 10.1107/S010827010500209X 15805616

[bb2] Akitsu, T., Komorita, S., Kushi, Y., Li, C., Kanehisa, N. & Kai, Y. (1997). *Bull. Chem. Soc. Jpn*, **70**, 821–827.

[bb3] Hu, X.-L., Xu, X.-Y., Wang, D.-Q. & Xu, T.-T. (2006). *Acta Cryst.* E**62**, m1922–m1923.

[bb4] Hu, X., Xu, X., Xu, T. & Wang, D. (2006). *Acta Cryst.* E**62**, m2221–m2223.

[bb5] Milne, P., Ho, M. & Weaver, D. F. (1999). *J. Mol. Struct. (THEOCHEM)*, **492**, 19–28.

[bb6] Sheldrick, G. M. (1996). *SADABS* University of Göttingen, Germany.

[bb7] Sheldrick, G. M. (2008). *Acta Cryst.* A**64**, 112–122. 10.1107/S0108767307043930 18156677

[bb8] Siemens (1996). *SMART* and *SAINT* Siemens Analytical X-ray Instruments Inc., Madison, Wisconsin, USA.

